# Molecular phylogeny of the higher and lower taxonomy of the *Fusarium *genus and differences in the evolutionary histories of multiple genes

**DOI:** 10.1186/1471-2148-11-322

**Published:** 2011-11-03

**Authors:** Maiko Watanabe, Takahiro Yonezawa, Ken-ichi Lee, Susumu Kumagai, Yoshiko Sugita-Konishi, Keiichi Goto, Yukiko Hara-Kudo

**Affiliations:** 1 Division of Microbiology, National Institute of Health Sciences, Kamiyoga 1-18-1, Setagaya-ku, Tokyo 158-8501, Japan; 2 School of Life Sciences, Fudan University, 220, Handan Rd. 200433, Shanghai, China; 3 Veterinary Medical Science, Graduate School of Agricultural and Life Sciences, The University of Tokyo, Yayoi 1-1-1, Bunkyo-ku, Tokyo 113-8657, Japan; 4 Food Research Laboratories, Mitsui Norin Co., Ltd., Miyabara 223-1, Fujieda-shi, Shizuoka 426-0133, Japan

## Abstract

**Background:**

Species of the *Fusarium *genus are important fungi which is associated with health hazards in human and animals. The taxonomy of this genus has been a subject of controversy for many years. Although many researchers have applied molecular phylogenetic analysis to examine the taxonomy of *Fusarium *species, their phylogenetic relationships remain unclear only few comprehensive phylogenetic analyses of the *Fusarium *genus and a lack of suitable nucleotides and amino acid substitution rates. A previous stugy with whole genome comparison among *Fusairum *species revealed the possibility that each gene in *Fusarium *genomes has a unique evolutionary history, and such gene may bring difficulty to the reconstruction of phylogenetic tree of *Fusarium*. There is a need not only to check substitution rates of genes but also to perform the exact evaluation of each gene-evolution.

**Results:**

We performed phylogenetic analyses based on the nucleotide sequences of the rDNA cluster region (rDNA cluster), and the β-tubulin gene (*β-tub*), the elongation factor 1α gene (*EF-1α*), and the aminoadipate reductase gene (*lys2*). Although incongruence of the tree topologies between *lys2 *and the other genes was detected, all genes supported the classification of *Fusarium *species into 7 major clades, I to VII. To obtain a reliable phylogeny for *Fusarium *species, we excluded the *lys2 *sequences from our dataset, and re-constructed a maximum likelihood (ML) tree based on the combined data of the rDNA cluster, *β-tub*, and *EF-1α*. Our ML tree indicated some interesting relationships in the higher and lower taxa of *Fusarium *species and related genera. Moreover, we observed a novel evolutionary history of *lys2*. We suggest that the unique tree topologies of *lys2 *are not due to an analytical artefact, but due to differences in the evolutionary history of genomes caused by positive selection of particular lineages.

**Conclusion:**

This study showed the reliable species tree of the higher and lower taxonomy in the lineage of the *Fusarium *genus. Our ML tree clearly indicated 7 major clades within the *Fusarium *genus. Furthermore, this study reported differences in the evolutionary histories among multiple genes within this genus for the first time.

## Background

Species of the *Fusarium *genus are well-known important plant pathogens, and are mycotoxin producers which are associated with human and animal health hazards [1,2]. *Fusarium *species are well-researched in many fields, such as molecular biology, ecology, phytopathology, medical-mycology, toxicology, and others. One problem commonly encountered by researchers interested in *Fusarium *species is the precise taxonomic system of the genus. In general, species are recognized on the basis of the morphological species concept, the biological species concept, the phylogenetic species concept or a combination of these [3]. Current classification schemes of fungi are exclusively based on the morphological species concept, and identification of the species primarily involves the use of morphological characteristics [4]. Although traditional taxonomic systems for the *Fusarium *genus have been proposed based on the morphological species concept, the taxonomy of this genus has been debated for many years [5-10]. Recently, many researchers have applied molecular phylogenetic analysis to examine the taxonomy of *Fusarium *species, and have proposed new taxonomic systems based on the phylogenetic species concept. However, many phylogenetic relationships remain unclear as only few comprehensive phylogenetic analyses of the *Fusarium *genus have been performed. Moreover, some previous studies have reported phylogenetic trees with a low resolution, especially in the deep lineages, due to a lack of suitable nucleotide and amino acid substitution rates and other factors.

Recently, whole genome comparison among four *Fusairum *species revealed the drastic genome-evolution such as the horizontal gene transfer in *Fusarium *genomes [11]. Therefore, it is possible for each gene in *Fusarium *genomes to have a unique evolutionary history, and it is necessary to perform exact evaluation of the evolutionary processes of each gene. Moreover, a unique evolutionary history of each gene in *Fusarium *genomes may bring difficulty to the reconstruction of phylogenetic tree among *Fusarium *species as mentioned above. There is a need not only to check the nucleotide and amino acid substitution rates of genes but also to perform the exact evaluation of each gene-evolution. Then, we should select suitable genes providing information for phylogenetic inference about both the higher and lower taxa in the *Fusarium *tree.

Previously, some parts of the rDNA cluster region, *β-tub*, and *EF-1α *have been used as genetic markers for the phylogenetic analysis of fungal taxonomic groups, including *Fusarium *species [12-14]. Furthermore, it has been reported that the *lys2 *may be a good phylogenetic marker for inferring relationships among fungal genera [15] and among species of the *Byssochlamys *genus and related genera [16]. In this study, we evaluated holistically the rDNA cluster region, *β-tub*, *EF-1α*, and *lys2 *as markers to infer the reliable species tree of *Fusarium *species, and re-constructed the phylogenetic tree using the maximum likelihood method.

## Results and discussions

### Seven major clades of the *Fusarium *genus and the incongruence of gene trees

**Figure 1:**
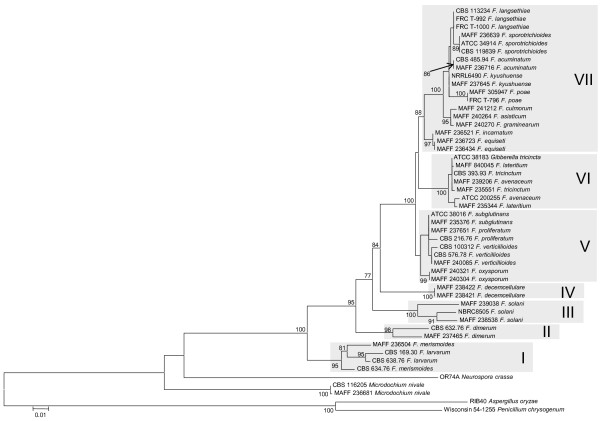
**Maximum likelihood trees for the *Fusarium *genus and related genera inferred from the rDNA cluster including 18S rDNA, ITS1, 5.8S rDNA and 28S rDNA**. The GTR + I + Γ model was used as the model for nucleotide substitution. Branch lengths are proportional to the estimated number of nucleotide substitutions. The BP values over 75% are displayed on the nodes (BP; 1000 replicates).

**Figure 2:**
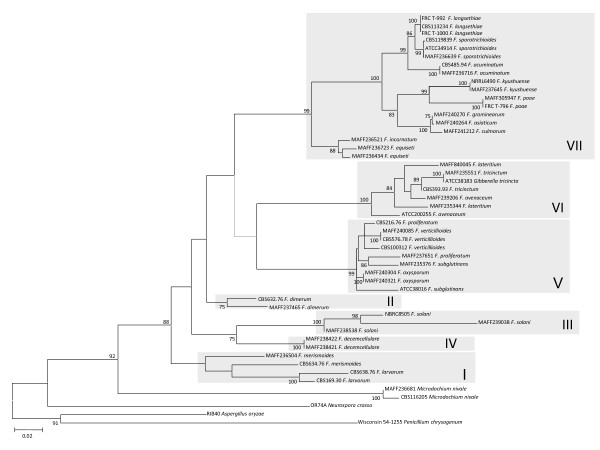
**Maximum likelihood trees for the *Fusarium *genus and related genera inferred from *β-tub***. The GTR + I + Γ model was used as the model for nucleotide substitution. Branch lengths are proportional to the estimated number of nucleotide substitutions. Each codon position was analysed separately. The BP values over 75% are displayed on the nodes (BP; 1000 replicates).

**Figure 3:**
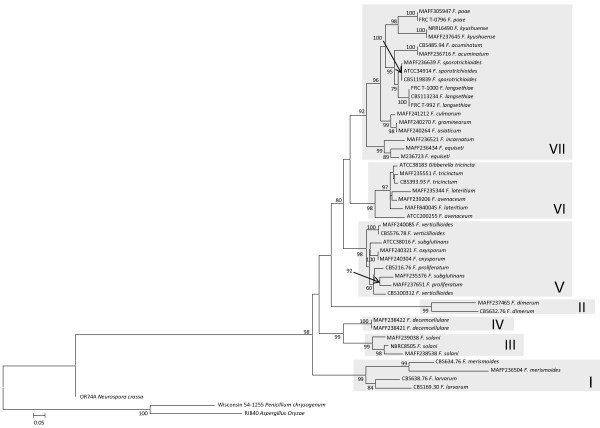
**Maximum likelihood trees for the *Fusarium *genus and related genera inferred from *EF-1α***. The GTR + I + Γ model was used as the model for nucleotide substitution. Branch lengths are proportional to the estimated number of nucleotide substitutions. Each codon position was analysed separately. The BP values over 75% are displayed on the nodes (BP; 1000 replicates).

**Figure 4:**
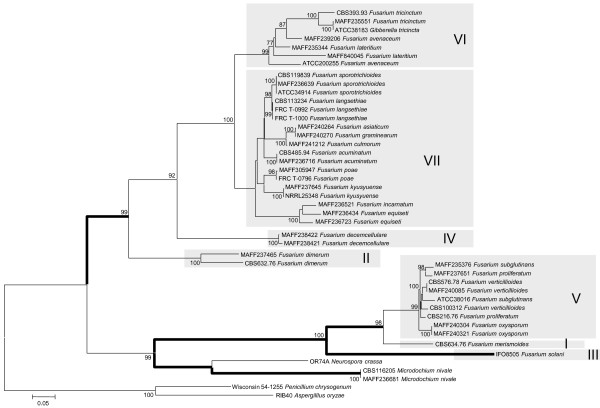
**Maximum likelihood trees for the *Fusarium *genus and related genera inferred from *lys2***. The GTR + I + Γ model was used as the model for nucleotide substitution. Branch lengths are proportional to the estimated number of nucleotide substitutions. Each codon position was analysed separately. The BP values over 75% are displayed on the nodes (BP; 1000 replicates). The branches with bold lines indicate the lineages in which positive selection has occurred with the p-value under the null hypothesis that the *ω *(synonymous substitutions per synonymous site/non-synonymous substitutions per non-synonymous site) of the positively selected sites is equal to 1.0. (*p *< 0.001).

The ML trees inferred from each of the concatenated parts of the rRNA cluster region (rDNA cluster), E-tub EF-1 D and lys2 are displayed in Figures 1, 2, 3 and 4, respectively. The tree topologies of the gene sequences were not consistent with each other. However, all of the gene trees supported the classification of *Fusarium *species into 7 major clades, namely, clades I to VII. Most of the support values for these clades were very high (more than 95% bootstrap value; BP), with the exceptions of clade I (71% BP) and clade II (75% BP) of the *β-tub *ML tree, clade V (88% BP) and clade VII (55% BP) of the rDNA cluster ML tree, and clade VII (<50% BP) of the *lys2 *ML tree. Our ML tree clearly indicated 7 major clades within the *Fusarium *genus for the first time.

Many of taxonomic studies based on morphological characters have reported that some "sections", including closely related species, share some "synapomorphic" character states. Clade I consists of *F. larvarum *and *F. merismoides *which belong to different "sections", namely, *Eupionnotes *and *Arachnites*, respectively. Although the *β-tub *and *EF-1α *sequences supported the monophyly of *F. merismoides*, the rDNA cluster supported a paraphyletic relationship for this species. Clades II, III, and IV consist of single species, namely, *F. dimerum, F. solani*, and *F. decemcellulare*, respectively. Clade V contains 2 "sections": *Elegans*, which consists of *F. oxysporum*, and *Liseola*, which consists of *F. subglutinans, F. proliferatum and F. verticillioides*. Clade VI consists of *F. lateritium, F. avenaceum*, and *F. tricinctum*, which belong to different "sections", namely, *Lateritium, Roseum*, and *Sporotrichiella *respectively. The paraphyly of *F. avenaceum *and *F. lateritium *was supported by all the genes. Clade VII contains 4 "sections" with 9 species: *Eupionnotes *consisting of *F. incarnatum, Gibbosum *consisting of *F. equiseti *and *F. acuminatum, Discolor *consisting of *F. graminearum *and *F. culmorum*, and *Sporotrichiella *consisting of *F. poae, F. kyusyuense, F. sporotrichioides*, and *F. langsethiae*. Our ML trees based on each of the rDNA cluster and the 3 genes indicate that the species in each of the clades (I to VII) have close relationships with each other.

Some taxonomic groups which were not previously identified using the morphological species concept, have already revealed by molecular phylogenetic analyses previously reported. O'Donnell et al. [14] showed that there are species complexes including more than 2 species, such as the *Gibberella fujikuroi *species complex. O'Donnell et al. [14] and O'Donnell and Cigelnik [14,17] showed that some sections proposed by morphological studies form paraphyletic or polyphyletic groups, such as *Sporotrichiella *and *Discolor*. This study supported their results. Moreover, our results indicated some new taxonomic groups, such as clade I and VI. The close relationships in two clades are discussed in the paragraph of "Verification of monophyly of the sections and species of the *Fusarium *genus", as described below.

### Evaluation of incongruence of the gene trees

The phylogenetic relationships among clades I to VII differ from each other, as described above. To evaluate this incongruence, we compared the log-likelihood scores of the relationships among clades I to VII (Figure 1) (see Additional file 1). The absolute values of the log likelihood scores of the ML trees, and the differences between the log-likelihood scores of the ML tree ± 1SE and of the alternative trees are displayed in Table 1. It can be concluded that. no significant differences were observed between the *EF-1α*, rDNA cluster, and *β-tub *ML trees, but all of these differed from the *lys2 *ML tree (p-value of the SH test <0.001).

**Table 1:** The comparison of the tree topologies for relationships of clades I to VII based on each gene

	The difference of the log-likelihood score from ML tree ± SD			
	
Topology	*β-tub*	*EF-1α*	rDNA cluster	*lys2*
ML tree of/*β-tub*	<-4585.10 >	-1.67 ± 1.81	-9.62 ± 5.69	-571.47 ± 40.65^a^
ML tree of *EF-1 α*	-4.12 ± 4.24	<-8781.88 >	-7.28 ± 4.93	-512.43 ± 43.93^a^
ML tree of rDNA cluster	-16.34 ± 7.73	-19.78 ± 8.29	<-4965.10 >	-514.52 ± 42.25^a^
ML tree of *lys2*	-33.97 ± 12.45^a^	-78.40 ± 16.91^a^	-91.24 ± 19.33^a^	<-9803.46 >

Angled parenthes: the absolute value of the log likelihood score of the ML tree.

^a^Significantly smaller score of the log-likelihood than that of the ML tree by the SH test.

What is the reason for the differences in tree topology observed among the genes? One possibility is that the tree inference is an artefact. Another possibility is that the genes have different evolutionary histories. In the former case, the main factors which can lead to misleading tree inferences include (1) long-branch attraction, (2) composition bias of nucleotide and amino acid, and (3) convergent evolution.

Long-branch attraction mainly occurs as a consequence of rapidly evolving sites, and removal of such sites from the analysis can reduce the effects of long-branch attraction [18]. Accordingly, we excluded the fast evolving sites of *lys2*. After the 3^rd ^codon position sites or synonymous substitution sites had been excluded, the tree topology remained essentially the same as the ML tree displayed in Figure 4. Furthermore, even when the ML tree was inferred based upon the 2^nd ^codon positions only, the tree topology remained essentially the same as the ML tree displayed in Figure 4. Therefore, the possibility of long-branch attraction is unlikely.

Extreme composition bias of nucleotides and amino acids can strongly mislead tree inference [19,20]. Therefore, we examined composition bias of *lys2 *nucleotides and amino acids in the lineages within our dataset. However, no composition bias was observed in the amino acid sequences or the combined 1^st^/2^nd ^codon position sites. Composition bias of the 3^rd ^codon position sites was detected in *F. lateritium *(MAFF235344 and MAFF840045), and of the combined 1^st^, 2^nd ^and 3^rd ^codon position sites in *F. tricinctum *(CBS393.93, ATCC38183, and MAFF235551). These species both belong to clade VI. Therefore, it is unlikely that nucleotide composition bias can explain the phylogenetically "misleading" observed among clades I to VII in ML trees displayed in Figures 1, 2, 3 and 4.

Although the principal theory of molecular phylogenetics is based on the neutral theory of molecular evolution [21], convergent evolution is known to occur at the molecular level and can mislead the reconstruction of phylogenetic trees [22,23]. As natural selection generally acts at the level of amino acid sequences, synonymous substitutions are unlikely to be affected by convergence. Therefore, we inferred the phylogenetic tree using only the 3^rd ^codon positions because substitutions at these sites are mainly synonymous. However, the *lys2 *ML tree inferred only using the 3^rd ^codon positions was essentially the same as the tree presented in Figure 4 (data not displayed).

For these 3 reasons, it is unlikely that the incongruence of the *lys2 *ML tree and the other gene trees was due to an analytical artefact. Instead, the differences in tree topology may reflect differences in the evolutionary histories of the considered genes. Therefore, when we reconstructed the species tree, we excluded *lys2 *from the analysis.

### Evaluation of genetic markers for phylogenetic reconstruction

To accurately reconstruct the phylogenetic tree, we selected the genes which displayed an adequate evolutionary rate. ML trees based on each individual gene or the rDNA regions are displayed in Figures 2, 3 and 4 and additional files 2, 3, 4 and 5. The substitution rates of the 3 rDNA regions (5.8S, 18S, and 28S) were all slow. Although the substitution rate of ITS1 was faster than that of the rDNA genes, the sequence length was very short (101 bp). Therefore, the nucleotide sequences of each 4 rDNA regions were identical in several *Fusarium *species. When the 3 rDNA genes and ITS1 were combined in a cluster, we could distinguish most of the species from the nucleotide sequence data. However, since only small differences were observed among species, some resolutions among the species in the same clade were unclear (Figure 1). In contrast, the nucleotide substitution rates of the protein coding genes, namely, *β-tub, EF-1α*, and *lys2*, were rapid, and each of these genes had a high resolution for the relationship among conspecific strains or closely related species in the same clade of I to VII (the lower taxa) (Figures 2, 3 and). However, in the cases of *β-tub and EF-1α*, the alignment of amino acid sequences among *Fusarium *species indicated that almost all of the substitutions were singletons, and parsimonious informative sites were limited (data not shown). Therefore, almost all of the substitutions which occurred within the genomes of *Fusarium *species were synonymous.

**Figure 5:**
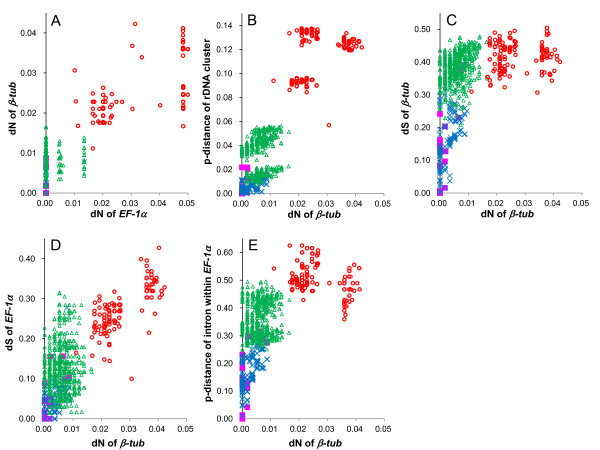
**Comparisons of the evolutionary distances for each gene**. Number of non-synonymous substitutions per non-synonymous site (dN) plotted against the number of synonymous substitutions per synonymous site (dS) with all of the strains in this study. In all graphs, we divided the distances into 4 groups; the pink square, between conspecific *Fusarium-stra*in*s: *the blue cross-mark, between *Fusarium-*strains of different species in the same clade of I to VII: the green triangle, between *Fusarium-*strains in different clades of I to VII: the red circle, between strains of *Fusarium *species and other genera. Panel A: non-synonymous substitution in *β-tub *vs. non-synonymous substitution in *EF-1α; *panel B: nucleotide substitution of rDNA genes vs. non-synonymous substitution in *β-tub*, panel C: synonymous substitution in *β-tub *vs. non-synonymous substitution in *β-tub*, panel D: synonymous substitution in *EF-1α *vs. non-synonymous substitution in *β-tub*, panel E: nucleotide substitution of the intron of *EF-1α*. vs. non-synonymous substitution in *β-tub*.

Pairwise comparisons of the substitution distances for the nucleotide and amino acid sequences are displayed in Figure 5 (Additional file 6). The distances in these graphs were divided into 4 groups; between conspecific *Fusarium*-strains, between *Fusarium*-strains of different species in the same clade of I to VII, between *Fusarium-*strains in different clades of I to VII, and between strains of *Fusarium *species and other genera. As for the non-synonymous substitutions of *β-tub *and *EF-1α*, the numbers of non-synonymous substitutions in these genes were too few to reconstruct the phylogenetic relationships especially among clades I to VII and among *Fusarim *and its related genera (the higher taxa) in the lineage of the *Fusarium *genus (Figure 5A). The other 4 regions, namely, the rDNA cluster, the synonymous substitutions of *β-tub *and *EF-1α*, and the introns within *EF-1α*, displayed the graphs forms split up into 2 groups: the synonymous substitutions of *β-tub*/the introns within *EF-1α *and the rDNA cluster/the synonymous substitutions of *EF-1α *(Figure 5). The graphs of the former group (Figures 5C and 5E) were almost parallel to the × axis for the higher taxa. This implies that these 2 regions are completely saturated in the case of examining among the higher. However, these substitutions are useful for examining among the lower taxa. The graphs of the latter group (Figures 5B and 5D) are not linear but still ever-increasing, and the substitution rates of this group are not so slow. This result suggests that these substitutions can provide information for phylogenetic reconstruction for both the higher and lower taxa.

One of the aims of this study was to provide a comprehensive description of the phylogenetic relationships among *Fusarium *species and closely related species, including both the higher and lower taxa. Therefore, we require information obtained from multiple substitutions in multiple genes to reconstruct the phylogenetic tree. In this study, we considered all of the substitution information obtained from the rDNA cluster and the *β-tub *and *EF-1α *genes. Substitutions within *lys2 *were removed from our dataset because this locus is not suitable for the phylogenetic analysis of *Fusarium *and its related species. We had to modify the weighting of some nucleotides in the analysis because the information obtained from substitutions varied among sites with a partition model, as described in the materials and methods.

### Phylogenetic relationships among clades of the *Fusarium *genus

**Figure 6:**
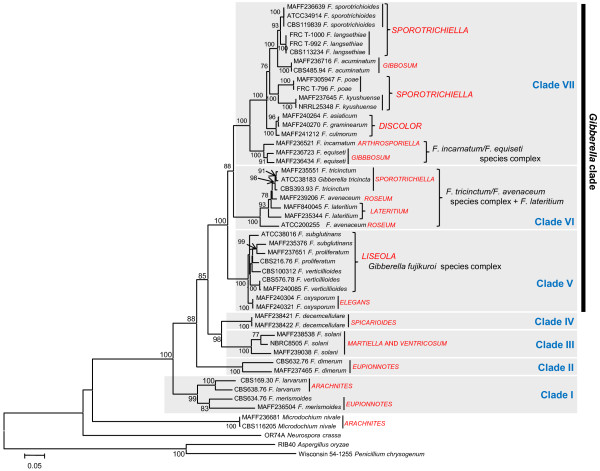
**Maximum likelihood tree of the *Fusarium *genus and related genera inferred from the combined sequences of the rDNA cluster and the *β-tub*, and *EF-α *genes**. Taking into account the different tempos and modes of nucleotide substitutions, all parameters of the substitution model were separately estimated for each gene using the GTR + I + Γ model. The branch lengths are proportional to the estimated number of nucleotide substitutions. For the protein-coding genes *β-tub *and *EF-1α*, each codon position was analysed separately. The bootstrap probability (BP; 1000 replicates) values over 75% are displayed on the nodes. Sections supported by morphological taxonomic systems are described, and include *Arachnites, Arthrosporiella, Discolor; Elegans, Eupionnotes, Gibbosum, Lateritium, Liseola, Martiella *and *Ventricosum, Roseum, Spicarioides*, and *Sporotrichiella*.

The ML tree based on the combined data from the rDNA cluster and the *β-tub *and *EF-1α *is displayed in Figure 6. The BP values of more than 75% are presented on the nodes. Our ML tree showed that *M. nivale *does not belong to any of the 7 major clades of the *Fusarium *species, and that this species, which is the common ancestor of *Fusarium *species and *N. crassa*, rapidly diverged during short term. Therefore, our analyses supported the previously described separation of *M. nivale *from the *Fusarium *genus [13,24].

Booth [5] proposed that closely related sections have the same teleomorph genus, and O'Donnell et al. [25] indicated that all *Fusarium *species with teleomorphs belonging to the *Gibberella *genus form a clade known as the *Gibberella *clade. In this study, all of the examined species with teleomorphs belonging to the *Gibberella *genus clustered in a super-clade consisting of clades V to VII (Figure 6). Our results thus support the classification of the *Gibberella *clade.

Marasas et al. [1] and Pitt and Hocking [2] were reviewed that many *Fusarium *species produced many kinds of mycotoxins and these toxins are associated with human and animal health hazard. One of the main mycotoxins which naturally pollute agricultural crops, is trichotecenes or fumonisins. Our ML tree indicated that most *Fusarium *species which produce trichothecenes, clustered in a super-clade consisting of clade VI and VII (Figure 6). This relationship suggested that a common ancestor had acquired the capacity to produce trichothecenes, and then some species might lose this capacity.

### Verification of monophyly of the sections and species of the *Fusarium *genus

In our ML tree (Figure 6), many sections, which have previously been defined based upon morphological characteristics, were not verified as monophyletic. In this analysis, only *Discolor *(100% BP) was supported by a BP value greater than 75%. Sections other than *Discolor*, namely *Arachnites, Eupionnotes, Gibbosum*, and *Sporotrichiella*, which include 2 or more species in this study, formed the paraphyletic and polyphyletic groups. These results are consistent with previous studies reporting that taxonomic groups, which were traditionally classified using the morphological species concept, are not always recovered in molecular phylogenetic analyses of the *Fusarium *genus. In particular, in the *Eupionnotes *section, species were assembled using the morphological species concept with section-specific characteristics such as a very slow growth rate on potato dextrose agar (PDA; Eiken) a yeast-like appearance due to the absence of aerial mycelia, absence of microconidia, and small macroconidia generally having only 1 to 2 septa [5,8]. Our results indicated that these characteristics are not synapomorphies shared among only species in this section, such as *F. dimerum *and *F. merismoides*. Moreover, morphological characteristics such as slow growth rate, color of colonies on PDA from below (white to tan), absence of microconidia, and small macroconidia appear in the basal lineages containing species of clades I and II, and *M. nivale*. Therefore, we consider these characteristics to be ancestral, and that they remain in species of the basal lineages as symplesiomorphies. It is difficult to distinguish among the synapomorphies, the symplesiomorphies, and convergent derived characters. Species recognition on the basis of morphology often comprises several species that are recognized on the basis of molecular phylogeny.

Although the morphological species concept does not reflect the phylogenetic tree of the *Fusarium *genus, this does not imply that morphological characteristics are not useful for identification and taxonomy. On the contrary, species recognition based on morphological characteristics is also useful for identifying unknown species because morphological characteristics can be widely applied to any species, not only those of the *Fusarium *genus but also other fungi [3,26]. *Fusarium *isolates can be initially classified on the basis of morphological similarity, with the awareness that sections are in fact a means of artificial grouping. Thus, it is still necessary to use recognition methods based upon morphological characteristics in combination with the phylogenetic recognition method.

Although our ML tree was constructed with a high resolution, the relationships among species that together form a complex could not be resolved. Previous molecular phylogenetic analyses have suggested that a species of the *Liseola *section, defined by traditional taxonomy based on the morphological species concept, actually includes multiple species, and that the re-defined novel species, which are recognized mainly by mating types, constitute the *Gibberella fujikuroi *species complex [15-28]. The ML tree obtained in this study (Figure 6) demonstrates convoluted, nested structures of species within the *Liseola *section. The monophylies of *F. subglutinans, F. proliferatum*, and *F. verticillioides*, which have been recognized by the traditional morphological species concept, were not recovered. When we recognize species based on the novel taxonomic system (see Table 2: species re-identified by molecular method), our ML tree could not resolved the phylogenetic relationships among re-identified species in the "*Gibberella fujikuroi *species complex" excluding the sister relationship between *F. phylophilum *and *F. fujikuroi*. This difficulty of resolving phylogenetic relationships in this complex is probably caused by rapid divergence events occurred in this species complex. It is implied by the shortened internal branches in this species complex in our ML tree.

**Table 2:** Strains of the genus *Fusarium *and *Fusarium*-related spece is used in this study

**Section**^**a**^	Species registered in resorce organization	**Species in tradittional taxonomic system**^**a**^	**Species re-identificated by molecular method**^**b**^	**Strain No**.
*Arachnites*	*F. larvarum*	*F. larvarum*	Not identified	CBS^c ^169.30 CBS 638.76 (Isotype strain)
	
	*Microdochium nivale*	*F. nivale*	Not identified	CBS 116205 (Isotype strain) MAFF^d ^236681

*Arthrosporiella*	*F. incarnatum*	*F. semitectum*	*F. incarnatum-equiseti species complex*	MAFF 236521

	*F. culmorum*	*F. culmorum*	*F. cerealis*	MAFF 241212
	
*Discolor*	*F. asiaticum*	*F. graminearum*	*F. asiaticum*	MAFF 240264
			
	*F. graminearum*		*F. graminearum*	MAFF 240270

*Elegans*	*F. oxysporum*	*F. oxysporum*	*F. oxysporum species complex*	MAFF 240304 MAFF 240321

	*F. dimerum*	*F. dimerum*	*F. lunatum*	CBS 632.76 (Neotype strain)
			
*Eupionnotes*			*F. penzigii*	MAFF 237465
	
	*F. merismoides*	*F. merismoides*	Not identified	CBS 634.76 (Type strain) MAFF 236504

*Gibbosum*	*F. equiseti*	*F. equiseti*	*F. incarnatum-equiseti species complex*	MAFF 236434 MAFF 236723
	
	*F. acuminatum*	*F. acuminatum*	*F. armeniacum*	CBS 485.94 MAFF 236716

*Lateritium*	*F. lateritium*	*F. lateritium*	*F. lateritium*	MAFF 235344
			
			Not identified	MAFF 840045

	*F. proliferatum*	*F. proliferatum*	*F. phylophilum*	CBS 216.76 (Type strain)
			
			*F. fujikuroi*	MAFF 237651
	
*Liseola*	*F. subglutinans*	*F. subglutinans*	*F. subglutinans*	ATCC 38016
			
			*F. sacchari*	MAFF 235376
	
			*F. verticillioides*	CBS 576.78 (Epitype strain)
			
	*F. verticillioides*	*F. moniliforme*	*F. thapsinum*	CBS 100312
			
			*F. verticillioides*	MAFF 240085

*Martiella-Ventricosum*	*F. solani*	*F. solani*	*F. solani species complex*	MAFF 238538 MAFF 239038 NBRC^f ^8505

*Roseum*	*F. avenaceum*	*F. avenaceum*	*F. nurragi*	ATCC 200255 (Type strain)
			
			Not identified	MAFF 239206

*Spicarioides*	*F. decemcellulare*	*F. decemcellulare*	Not identified	MAFF 238421 MAFF 238422

	*F. kyushuense*	Not described	*F. kyushuense*	MAFF 237645 (Ex holotype strain) NRRL^g ^6490 (Type strain)
	
	*F. langsethiae*	Not described	*F. langsethiae*	CBS 113234 (Holotype strain) FRC^h ^T-0992 FRC T-1000
	
*Sporotrichiella*	*F. poae*	*F. poae*	Not identified	FRC T-0796 MAFF 305947
	
	*F. sporotrichioides*	*F. sporotrichioides*	Not identified	ATCC 34914 CBS 119839 MAFF 236639
	
	*Gibberella tricincta*			ATCC 38183 (Type strain)
				
	*F. tricinctum*	*F. tricinctum*	*F. tricinctum species complex*	CBS 393.93 (Epitype strain) MAFF 235551

^a^Nelson, Toussoun, and Marasas, 1983. *Fusarium *species- An Illustrated Manual for Identification.

^b^Sequence homology compared with data of EF-1*α *in the *Fusarium *ID (http://isolate.fusariumdb.org/index.php).

^c^Centraalbureau voor Schimmelcultures.

^d^Ministry of Agriculture, Forestry and Fisheries.

^e^American Type Culture Collection.

^f^National Institute of Technology and Evaluation, Biological Resource Center.

^g^Agricultural Research Service Culture Collection in United States Department of Agriculture.

^h^Fusarium Research Center in The Pennsylvania State University.

Furthermore, our study detected an additional intermingled, nested structure as a species complex containing the *F. avenaceum/F. tricinctum/F. lateritium *clade (clade VI in Figures 1 and 6). Regarding *F. avenaceum*, the affinities with *F. acuminatum *have been suggested by previous morphological and molecular studies [8,28]. However, other molecular studies have suggested that *F. avenaceum *is more closely related to *F. tricinctum *than to *F. acuminatum *[29,30]. Moreover, the ML tree in this study suggested the presence of a *F. avenaceum*/*F. tricinctum*/*F. lateritium *clade (Figure 6). The phylogenetic hypothesis of a sister-species relationship between *F. avenaceum *and *F. acuminatum *was completely rejected by our ML tree in Figure 6. Further studies with more strains of each of the species within these complexes are required for the clarification of taxonomic ambiguities.

### Adaptive evolution of the lys2 gene within the *Fusarium *genus

The topology of the *lys2 *tree was very different from that of the other trees (Figure 1, 2, 3, 4 and Table 1), and it indicated that the *Fusarium *genus is paraphyletic. Interestingly, by further investigation using the branch-site model, we detected many branches which displayed evidence of positive selection (The p value of the likelihood ratio test <0.001), indicated by bold branches in our *lys2 *tree (Figure 4). Generally, the incongruence of the species trees and the species trees were caused by the following three reasons: (1) ancestral polymorphism and incomplete lineage sorting, (2) gene duplication, and (3) horizontal gene transfer. We briefly describe these three hypotheses respectively as well as the difficulties of them in the following paragraphs.

In support of the first hypothesis, there was the polymorphism in the ancestral population of the *Fusarium *genus and its related genera, and some alleles appeared to have been positively selected, and finally fixed in each lineage independently. However, we should assume that the ancestral polymorphism may have been maintained for a very long time, such as several hundred million years. For the 18S rDNA gene, the average number of nucleotide substitutions between *Fusarium *and *M. nivale *is 17.0 and that between *Fusarium *and *N. crassa *is 30.9. This is similar to the number of differences observed between the human and chicken 18S rDNA genes (24 substitutions), and these lineages are thought to have diverged approximately 320 million years ago [31]. In the other hand, the average numbers of the nucleotide substitutions among *Fusarium *species is 1.9. The large difference among three genera and the small difference within the *Fusarium *genus indicate that it had taken long time until the emergence of the latest common ancestor of the *Fusarium *genus after the split of three genus.

**Figure 7:**
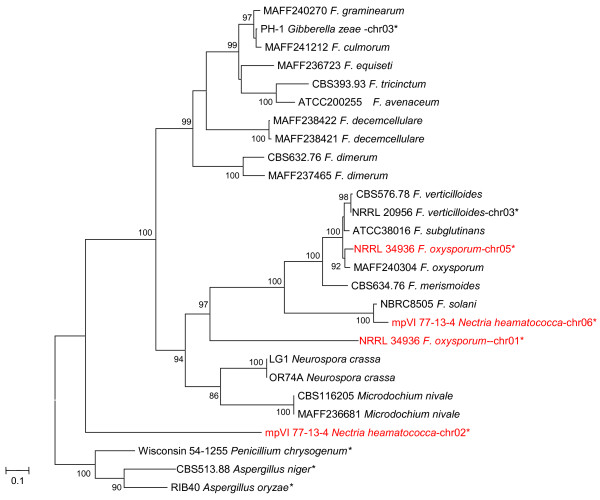
**Maximum likelihood tree inferred from *lys2***. The GTR + I + Γ model was used as the model of nucleotide substitution. The bootstrap probability (BP; 1000 replicated) values more than 75% are shown on the nodes. Branch lengths are proportional to the estimated number of nucleotide substitutions. The *lys2 *sequences of Wisconsin 54-1255 *Penicillium chrysogenum *(XM_002564615), CBS513.88 *Aspergillus niger *(XM_001401869), and RIB40 *Aspergillus oryzae *(XM_001820142) were used as outgroups. With the *Fusarium *dataset sequenced in this study, the *lys2 *sequences of PH-1 *Gibberella zeae*, NRRL 34936 *Fusarium oxysporum*, NRRL 20956 *Fusarium verticillioides*, mpVI 77-13-4 *Nectria haematococca *and *Neurospora crassa *were downloaded from the *Fusarium *Comparative Database, and also analysed. Data obtained from GenBank or were downloaded from the *Fusarium *Comparative Database are denoted by an asterisk. The species which have additional *lys2 *copies in their genomes are indicated in red.

The second hypothesis is that a gene duplication of lys2 event may have occurred in the common ancestor of the *Fusarium *genus and its related genera. Therefore, the amino acid sequences of these groups could radically change. In each lineage, 1 copy may have been positively selected, while the other may have become a non-functional pseudogene which was eventually purged from the genome. We found additional *lys2 *copies in the genomes of *F. oxysporum *and *Nectria haematococca *(anamorph; *F. solani*), but not in the genomes of *F. verticillioides *and *Gibberella zeae *(anamorph; *F. graminearum*), which were obtained from GenBank and the *Fusarium *Comparative Database (http://www.broadinstitute.org/annotation/genome/fusarium_group/MultiHome.html). We re-constructed the gene tree of *lys2 *with sequences from this study and from GenBank (Figure 7). The tree suggests that gene duplications may occur repetitively. However according to our analysis using the branch model, the additional *lys2 *gene copies may still be subject to purifying selection (data not shown). Therefore, it is unlikely that 1 copy will be completely purged from the genome in each lineage after the alternative copy has been positively selected.

The third hypothesis is that horizontal gene transfer has repeatedly occurred from the genome of 1 lineage to another. Horizontal gene transfer in the fungal genome has previously been observed among various fungal species including *F. oxysporum *[11,32]. We can infer when and where such events might have occurred during the evolutionary history of *lys2 *by comparing between the species tree (Figure 6) and the gene tree (Figure 4). The first horizontal gene transfer appears to have occurred from *N. crassa *(or *M. nivale*) to the *Fusarium *species in clade III. A second horizontal gene transfer appears to have occurred from clade III to clades I and V, respectively. The hypothesis that repetitive gene transfer events have occurred explains not only the positive selection but also the distantly related phylogenetic positions between the original *lys2 *gene and the additional *lys2 *copies found only in *F. oxysporum *and *N. haematococca *(Figure 7). However, the mechanism by which the new gene copy completely replaced the original copy in the hosts remains unclear.

Among these three hypotheses, the first hypothesis; ancestral polymorphism and incomplete lineage sorting, is unlikely. As mentioned above, although other two hypothesis can partially explain the incongruence of the gene trees and positive selections of *lys2*, the difficulties remain in all of the hypotheses. Hence, we cannot identify the reasons for them from our results.

The *lys2 *gene is fungus specific and is related to the synthesis of lysine [15]. Our results indicate that this gene has been subjected to positive selection within the *Fusarium *genus. Therefore, the metabolism of lysine is expected to be similar among the species within clades I, III, and V and within clades II, IV, VI, and VII. However, this should be confirmed using biochemical experiments. An et al. [15,33] and Watanabe et al. [16] did not report multiple copies of *lys2 *in the genomes of fungal genera such as *Aspergillus, Byssochlamys, Saccharomyces*, and others excluding *Fusarium*. Therefore, research and detection of other genera containing multiple copies of *lys2 *in their genomes is required to understand the diversity of lysine-metabolizing systems of fungi. At the same time, we should note the difficulties of the estimation of the *ω *ratios (the number of non-synonymous substitutions per non-synonymous site/the number of synonymous substitutions per synonymous site; dN/dS) in such a divergent taxon. The detection of the positive selection based on the *ω *ratios is highly sensitive to the saturation of the synonymous substituions. Since the relative evolutionary rate of the *lys2 *is high among the genetic markers used in this study [34], it is possible that the synonymous substitutions of *lys2 *were already saturated. However, the numbers of the multiple synonymous substitution at the same synonymous sites (multiple hits) were well estimated by the ML method using the codon substitution model, and ML method effectively correct the effect of multiple hits (Additional files 7 and 8). Moreover, w e applied the strict criterion to evaluate the statistical significance (p < 0.001) to completely exclude the possibility of the overestimation of the detection for the positive selection. Therefore, we could completely exclude the possibility that some of the detected positive selections were false-positive.

Further understanding of the evolutionary processes of *lys2 *and other genes is very important. To date, the full genome sequences of *Fusarium *are available for only 4 species of the *Gibberella *clade. The full genome sequences from other clades of *Fusarium *may elucidate the evolutionary processes of the genome, and such studies are currently in progress.

## Conclusion

This study reports the reliable phylogenetic tree of the higher and lower taxonomy of the lineage of the *Fusarium *genus. Our ML tree clearly indicates that there are 7 major clades containing *Fusarium *species. These clades were supported with high BP values in all of the phylogenetic trees based on single genes. Moreover, our results indicate the considerable differences in the evolutionary histories of multiple genes in the lineage of the *Fusarium *genus, particularly *lys2*.

## Methods

### Strains

The strains used in this study are listed in Table 2. We selected 24 species from the genus *Fusarium *and its related genera according to nomenclature system proposed by Nelson et al. [8]. This nomenclature is based on traditional species recognition methods. It is very simple and systematized taxonomy, and is widespread application in the field of identification of *Fusarium *isolates. To cover a wide range of taxonomic groups, we selected additional species referring molecular phylogenetic studies [35,36]. Each species includes one to three strains, and we tested a total of 47 strains. We further purified all *Fusarium *strains by the single-spore method [8].

### DNA extraction

We used two subcultures of each *Fusarium *and *Fusarium*-related species obtained by the single-spore method for sequencing. We checked their sequence identity between two subcultures to confirm purity of the strain. Samples were cultured on synthetic low nutrient agar (SNA; [26]) slant media supplemented with chloramphenicol 100 mg/l) at 25°C for 14 days. Mycelia and conidia from the slant culture were inoculated into 1 ml potato dextrose broth (Difco Laboratories) in a microtube, and were incubated at 25°C for 3 days. These fungal bodies were clumped by centrifugation at 18,000 × *g *for 10 min in a microtube. The genomic DNA was extracted from these pellets using the SDS method with minor modifications, as previously described [37].

### Amplification and sequencing of genes

The ribosomal RNA gene (rDNA) cluster region, including the 3' end of the 18S rDNA, the internal transcribed spacer region 1 (ITS1), the 5.8S rDNA, and the 5' end of the 28S rDNA, and the *β-tub, EF-1α*, and *lys2 *were selected as the regions for analysis. We performed amplification reactions with the primer pair for each gene using TaKaRa ExTaq (TaKaRa Bio Inc.), according to the manufacturer's instructions, in a thermal cycler (GeneAmp PCR System 9700; Applied Biosystems). The ITS1, 5.8S rDNA, and 5' end of the 28S rDNA were amplified in 1 fragment using the primer pair, ITS5 (5'-GGAAGTAAAAGTCGTAACAAGG-3'; [38])/NL4 (5'-GGTCCGTGTTTCAAGACGG-3'; [36]). For PCR amplification other than that for ITS1, 5.8S rDNA, and 28S rDNA, we used the following primer pairs: FF1 (5'-GTTAAAAAGCTCGTAGTTGAAC-3'; [39])/FR1 (5'-CTCTCAATCTGTCAATCCTTATT-3'; [39]) for 18S rDNA; Btu-F-F01 (5'-CAGACCGGTCAGTGCGTAA-3')/Btu-F-R01 (5'-TTGGGGTCGAACATCTGCT-3') for *β-tub; *EF-1 (5'-ATGGGTAAGGARGACAAGAC-3'; [40])/EF-2 (5'-GGARGTACCAGTSATCATGTT-3'; [40]) for *EF-1α; *and 2 primer pairs of Fulys2-F03mix (5'-CTTTGTTGGTGATGTTCTSA-3')/Fulys2-R01 (5'-TGGTAGGTCCGATATCGGT-3') and Fulys2-F04mix (5'- GCYATGGGDCARATYCTKGT -3')/Fulys2-R04mix (5'-CGGYTCYTCRTTRCGRTCTCT-3') for *lys2*. The primer pairs for *β-tub *and *lys2 *were designed based on sequences derived from primers used in previous studies, respectively [16,17]. They were designed to amplify the genes effectively. The PCR program consisted of an initial denaturing step at 94°C for 5 min, 35 amplification cycles, and an additional extending step at 72°C for 3 min. For the primer pairs FF1/FR1, ITS5/NL4, Fulys2-F03mix/Fulys2-R01, and Fulys2-F04mix/Fulys2-R04mix, the amplification cycles were 94°C for 30 s, 52°C for 40 s, and 72°C for 1 min and 10 s. For the primer pair Btu-F-F01/Btu-F-R01, the amplification cycles were 94°C for 30 s, 60°C for 40 s, and 72°C for 1 min. The PCR products were purified using ExoSap-IT (USB; Cleveland, OH). Dye labelling of the PCR products was performed with the same primers which were used for each gene in the amplification reactions, using the BigDye Terminator v. 3.1 Cycle Sequencing Kit (Applied Biosystems). For the PCR products from ITS5/NL4, we used the additional primer ITS3 (5'-GCATCGATGAAGAACGCAGC-3'; [38]). The labelled PCR products were ethanol precipitated according to the manufacturer's instructions, and then directly sequenced using the ABI 3130 analyzer (Applied Biosystems). The sequences were assembled using ATGC software (Genetyx Corporation). The *lys2 *gene of strains CBS 169.30, CBS 638.76, MAFF 236504, MAFF238538, and MAFF 239038, and the *EF-1*α gene of strains CBS 116205 and MAFF 236681 could not be amplified by PCR. The sequences determined in this study have been deposited in GenBank (accession nos. AA0000).

### Phylogenetic analysis based on DNA sequences

The nucleotide sequence datasets for each gene (18S rDNA, ITS1, 5.8S rDNA, 28S rDNA, *β-tub, EF-1α*, and *lys2*) were automatically aligned using the MUSCULE program [41]. Alignments were carefully checked visually and were manually modified; all ambiguous sections were excluded from the analysis. All intron regions of *β-tub *and *lys2 *were excluded, and we aligned only the exons. However, the introns for *EF-1α *comprised a relatively large proportion of our sequence data for this gene (63.8%), and these sections were retained for the analysis. We used *β-tub, EF-1α*, and *lys2 *exon sequences from several *Fusarium *and its related genus species, including PH-1 *Gibberella zeae*, NRRL 34936 *F. oxysporum*, and NRRL 20956 *F. verticillioides*, as references for the alignments. These sequences were downloaded from the *Fusarium *Comparative Database. The final lengths of the sequences are as follows: rDNA cluster (1314 bp: 18S rDNA = 509 bp, ITS1 = 101 bp, 5.8S rDNA = 159 bp, and 28S rDNA = 545 bp), *β-tub *(768 bp), *EF-1a *(804 bp), and *lys2 *(948 bp). Two Eurotiomycetes species, *Penicillium chrysogenum *Wisconsin 54-1255 (GenBank accession nos. XM_002559715, XM_002564615, and AM920418) and *Aspergillus oryzae *RIB40 (GenBank accession nos. XM_001825624, XM_001820142, and AP007172) were used as outgroups. The Sordariomycetes species *N. crassa *OR74A (GenBank accession nos. XM_952576, XM_960303 and FJ360521) was also used in the analysis to examine the monophyly of the *Fusarium *genus.

Phylogenetic trees were inferred using the ML method. This method has the corrective effect of multiple hits. The RAxML program ver. 7.0.3 [42] was used for the heuristic search.

Taking into account the different tempos and modes of the nucleotide substitutions as described in Figure 5, we separately estimated all parameters using the GTR + I + Γ substitution model for each partition. The partitions were as follows: rDNA region, ITS1 region, each codon site of *β-tub*, each codon site of *lys2*, introns of *EF-1α*, and each codon site of *EF-1α*. The branch lengths of each partition were estimated separately with independent model (see [43]). Since the RAxML program could not accurately estimate the branch lengths of *β-tub *by this method, we used the default option for this gene. To determine the confidence for the internal nodes, the rapid bootstrap method [42] was applied (1000 replications). The BASEML programs of PAML ver. 4.4 [44] were used for the exhaustive search. The GTR + Γ model was used, and all parameters of the substation model were separately estimated using the same partitions as mentioned above. The branch lengths were estimated using the proportional model [45]. To evaluate incongruence among the different gene trees, we compared the log-likelihood scores and their standard deviations by the Shimodaira-Hasegawa SH test [46], using the BASEML program. As we only focused on the relationships among major clades (seven clades of the *Fusarium *genus, *M. nivale *and *N. crassa*), we swapped the major clades of the ML trees for each gene one by one, and then compared the different tree topologies.

We compared the observed substitutions among genes, except for *lys2*, to evaluate the effects of saturation due to multiple hits. Pairwise comparisons of the observed number of synonymous and non-synonymous substitutions per site were calculated for all sequences of the 50 tested strains by Nei and Gojobori's method [47], without any correction, using MEGA ver. 5.0 software [48]. The p-distances of the rDNA cluster and the *EF-1α *introns were also calculated using MEGA ver. 5.0.

A *χ*^2 ^test was used to examine nucleotide and amino acid composition bias in particular lineages using TREE-PUZZLE ver 5.2 [49]. Composition bias was tested with datasets containing only the 3^rd ^codon position sites, the combined 1^st ^and 2^nd ^codon position sites, and the combined 1^st^, 2^nd^, and 3^rd ^codon position sites and amino acids.

### Detection of positive selection

Positive selection in particular lineages was detected by the branch-site model [50] using the CODEML program of PAML ver. 4.4. The statistical significance of positive selection was tested using likelihood ratio tests to compare the observed substitutions with the null hypothesis, which assumed that the ratio of non-synonymous rates/synonymous rates (*ω*) was equal to 1 [48].

## Authors' contributions

MW, GK, and YH-K conceived and designed the study. MW, YS-K, and YH-K collected the fungal strains. MW and K-IL performed the experiments. TY analyzed the data. MW, TY, and YH-K drafted manuscript and wrote the paper. SK, YS-K, K-IL and GK participated in the preparing the draft and revised the manuscript. All authors read and approved the final manuscript.

## Supplementary Material

Additional file 1 **Supplementary method S1**. Click here for file

Additional file 2 **Supplementary figure S1**. Maximum likelihood trees of the genus *Fusarium *and its related genera inferred from 18S rRNA gene (rDNA). Click here for file

Additional file 3 **Supplementary figure S2**. Maximum likelihood trees of the genus *Fusarium *and its related genera inferred from internal transcribed spacer region 1. Click here for file

Additional file 4 **Supplementary figure S3**. Maximum likelihood trees of the genus *Fusarium *and its related genera inferred from 5.8S rDNA. Click here for file

Additional file 5 **Supplementary figure S4**. Maximum likelihood trees of the genus *Fusarium *and its related genera inferred from 28S rDNA. The GTR + I + Γ model was used as the model of the nucleotide substitution. The nodal numbers indicate the bootstrap probability (BP; 1000 replicated). The branch lengths are proportional to the estimated number of nucleotide substitutions. The BP values more than 75% are shown on the nodes. Although the RAxML program inferred the ML tree of 28S rDNA including all 50 strains, it could not be summarized nodal BPs. Therefore, all identical sequences were excluded and remaining 31 sequences were used for the estimation of nodal BPs. Click here for file

Additional file 6 **Supplementary method S2**. Click here for file

Additional file 7 **Supplementary method S3**. Click here for file

Additional file 8 **Supplementary figure S5**. Comparisons of the evolutionary distances by two methods. Number of non-synonymous substitutions per non-per synonymous site (dS). Panel A: Distance method modified Nei-Gojorbori(JC) k = 2.5; panel B: ML method the codon model (pairwise). The estimated synonymous substitution reached plateau from the analysis by Nei Gojobori method. However there was no such tendency from the analysis by the maximum likelihood method. It means that although there were many multiple substitutions at the synonymmous sites, the maximum likelihood effectively correct the numbers of the multiple substitutions. Click here for file
