# Supplementary material for: Molecular phylogeny of the higher and lower taxonomy of the Fusarium genus and differences in the evolutionary histories of multiple genes
Source: BMC Evol Biol. 2011 Nov 3;11:322. doi: 10.1186/1471-2148-11-322 (PMC3270093; doi:10.1186/1471-2148-11-322)
Supplement: Additional file 1 — Supplementary method S1. [file 1471-2148-11-322-S1.DOC]

**Additional file 1 – Supplementary method S1.**

The relationships within each clade were fixed according to the ML trees for each gene (Figure 1), and only the relationships among clades I to VII and the related genera of *Microdochium* and *Neurospora* were compared. The relationships among clades I to VII together with *Microdochium nivale* (M) and *Neurospora crassa* (N) are as follows: the rDNA cluster ML tree, (M,(N,(I,(II,(III,(IV,(V,(VI,VII)))))))); β*-tub* ML tree, (N,(M,(I,((III,IV),(II,((V,VI),VII)))))); *EF-1*α ML tree, (N,(I,((III,IV),(II,(V,(VI,VII)))))); and *lys2* ML tree, ((II,(IV,(VI,VII))),(((I,V),III),(N,M))).
