# Supplementary material for: Molecular phylogeny of the higher and lower taxonomy of the Fusarium genus and differences in the evolutionary histories of multiple genes
Source: BMC Evol Biol. 2011 Nov 3;11:322. doi: 10.1186/1471-2148-11-322 (PMC3270093; doi:10.1186/1471-2148-11-322)
Supplement: Additional file 2 — Supplementary figure S1. Maximum likelihood trees of the genus Fusarium and its related genera inferred from 18S rRNA gene (rDNA). [file 1471-2148-11-322-S2.PPT]

## Slide 1
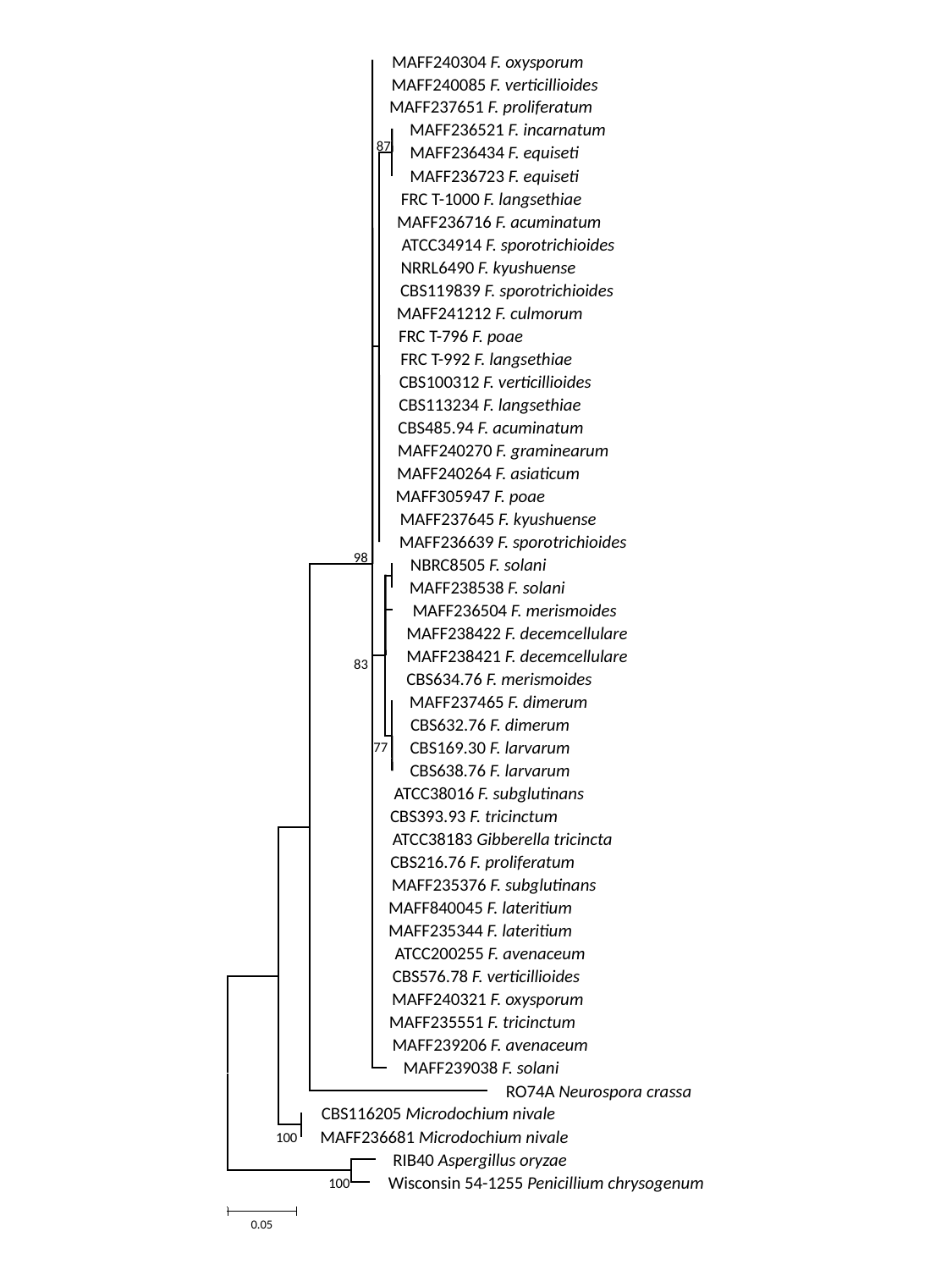

MAFF240304 F. oxysporum
 MAFF240085 F. verticillioides
 MAFF237651 F. proliferatum
 MAFF236521 F. incarnatum
87
 MAFF236434 F. equiseti
 MAFF236723 F. equiseti
 FRC T-1000 F. langsethiae
 MAFF236716 F. acuminatum
 ATCC34914 F. sporotrichioides
 NRRL6490 F. kyushuense
 CBS119839 F. sporotrichioides
 MAFF241212 F. culmorum
 FRC T-796 F. poae
 FRC T-992 F. langsethiae
 CBS100312 F. verticillioides
 CBS113234 F. langsethiae
 CBS485.94 F. acuminatum
 MAFF240270 F. graminearum
 MAFF240264 F. asiaticum
 MAFF305947 F. poae
 MAFF237645 F. kyushuense
 MAFF236639 F. sporotrichioides
98
 NBRC8505 F. solani
 MAFF238538 F. solani
 MAFF236504 F. merismoides
 MAFF238422 F. decemcellulare
 MAFF238421 F. decemcellulare
83
 CBS634.76 F. merismoides
 MAFF237465 F. dimerum
 CBS632.76 F. dimerum
 CBS169.30 F. larvarum
77
 CBS638.76 F. larvarum
 ATCC38016 F. subglutinans
 CBS393.93 F. tricinctum
 ATCC38183 Gibberella tricincta
 CBS216.76 F. proliferatum
 MAFF235376 F. subglutinans
 MAFF840045 F. lateritium
 MAFF235344 F. lateritium
 ATCC200255 F. avenaceum
 CBS576.78 F. verticillioides
 MAFF240321 F. oxysporum
 MAFF235551 F. tricinctum
 MAFF239206 F. avenaceum
 MAFF239038 F. solani
 RO74A Neurospora crassa
 CBS116205 Microdochium nivale
 MAFF236681 Microdochium nivale
100
 RIB40 Aspergillus oryzae
Wisconsin 54-1255 Penicillium chrysogenum
100
0.05
