# Supplementary material for: Molecular phylogeny of the higher and lower taxonomy of the Fusarium genus and differences in the evolutionary histories of multiple genes
Source: BMC Evol Biol. 2011 Nov 3;11:322. doi: 10.1186/1471-2148-11-322 (PMC3270093; doi:10.1186/1471-2148-11-322)
Supplement: Additional file 4 — Supplementary figure S3. Maximum likelihood trees of the genus Fusarium and its related genera inferred from 5.8S rDNA. [file 1471-2148-11-322-S4.PPT]

## Slide 1
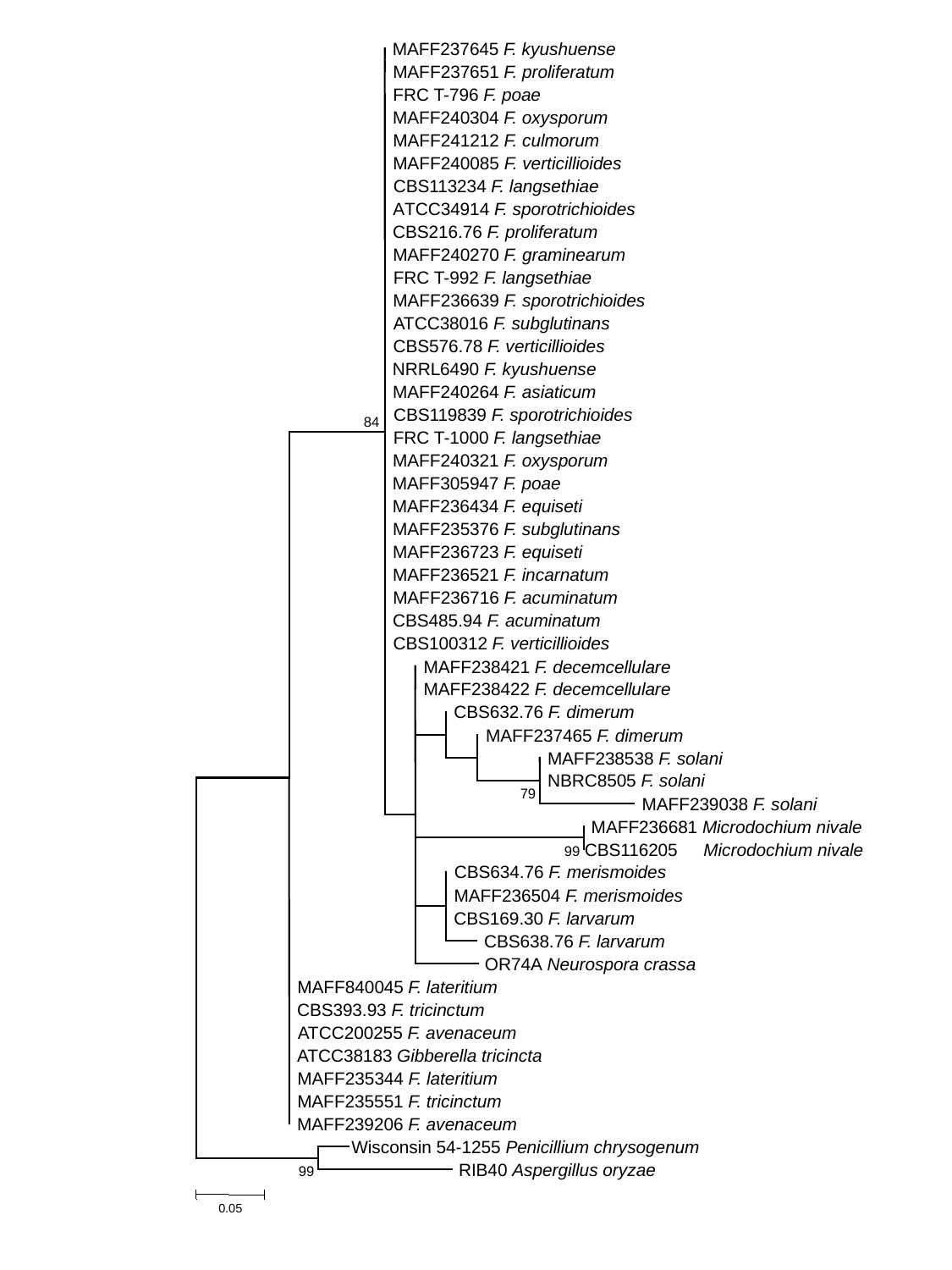

MAFF237645 F. kyushuense
 MAFF237651 F. proliferatum
 FRC T-796 F. poae
 MAFF240304 F. oxysporum
 MAFF241212 F. culmorum
 MAFF240085 F. verticillioides
 CBS113234 F. langsethiae
 ATCC34914 F. sporotrichioides
 CBS216.76 F. proliferatum
 MAFF240270 F. graminearum
 FRC T-992 F. langsethiae
 MAFF236639 F. sporotrichioides
 ATCC38016 F. subglutinans
 CBS576.78 F. verticillioides
 NRRL6490 F. kyushuense
 MAFF240264 F. asiaticum
 CBS119839 F. sporotrichioides
84
 FRC T-1000 F. langsethiae
 MAFF240321 F. oxysporum
 MAFF305947 F. poae
 MAFF236434 F. equiseti
 MAFF235376 F. subglutinans
 MAFF236723 F. equiseti
 MAFF236521 F. incarnatum
 MAFF236716 F. acuminatum
 CBS485.94 F. acuminatum
 CBS100312 F. verticillioides
 MAFF238421 F. decemcellulare
 MAFF238422 F. decemcellulare
 CBS632.76 F. dimerum
 MAFF237465 F. dimerum
 MAFF238538 F. solani
 NBRC8505 F. solani
79
 MAFF239038 F. solani
 MAFF236681 Microdochium nivale
 CBS116205　Microdochium nivale
99
 CBS634.76 F. merismoides
 MAFF236504 F. merismoides
 CBS169.30 F. larvarum
 CBS638.76 F. larvarum
 OR74A Neurospora crassa
 MAFF840045 F. lateritium
 CBS393.93 F. tricinctum
 ATCC200255 F. avenaceum
 ATCC38183 Gibberella tricincta
 MAFF235344 F. lateritium
 MAFF235551 F. tricinctum
 MAFF239206 F. avenaceum
Wisconsin 54-1255 Penicillium chrysogenum
 RIB40 Aspergillus oryzae
99
0.05
