# Supplementary material for: Molecular phylogeny of the higher and lower taxonomy of the Fusarium genus and differences in the evolutionary histories of multiple genes
Source: BMC Evol Biol. 2011 Nov 3;11:322. doi: 10.1186/1471-2148-11-322 (PMC3270093; doi:10.1186/1471-2148-11-322)
Supplement: Additional file 5 — Supplementary figure S4. Maximum likelihood trees of the genus Fusarium and its related genera inferred from 28S rDNA. The GTR + I + Γ model was used as the model of the nucleotide substitution. The nodal numbers indicate the bootstrap probability (BP; 1000 replicated). The branch lengths are proportional to the estimated number of nucleotide substitutions. The BP values more than 75% are shown on the nodes. Although the RAxML program inferred the ML tree of 28S rDNA including all 50 strains, it could not be summarized nodal BPs. Therefore, all identical sequences were excluded and remaining 31 sequences were used for the estimation of nodal BPs. [file 1471-2148-11-322-S5.PPT]

## Slide 1
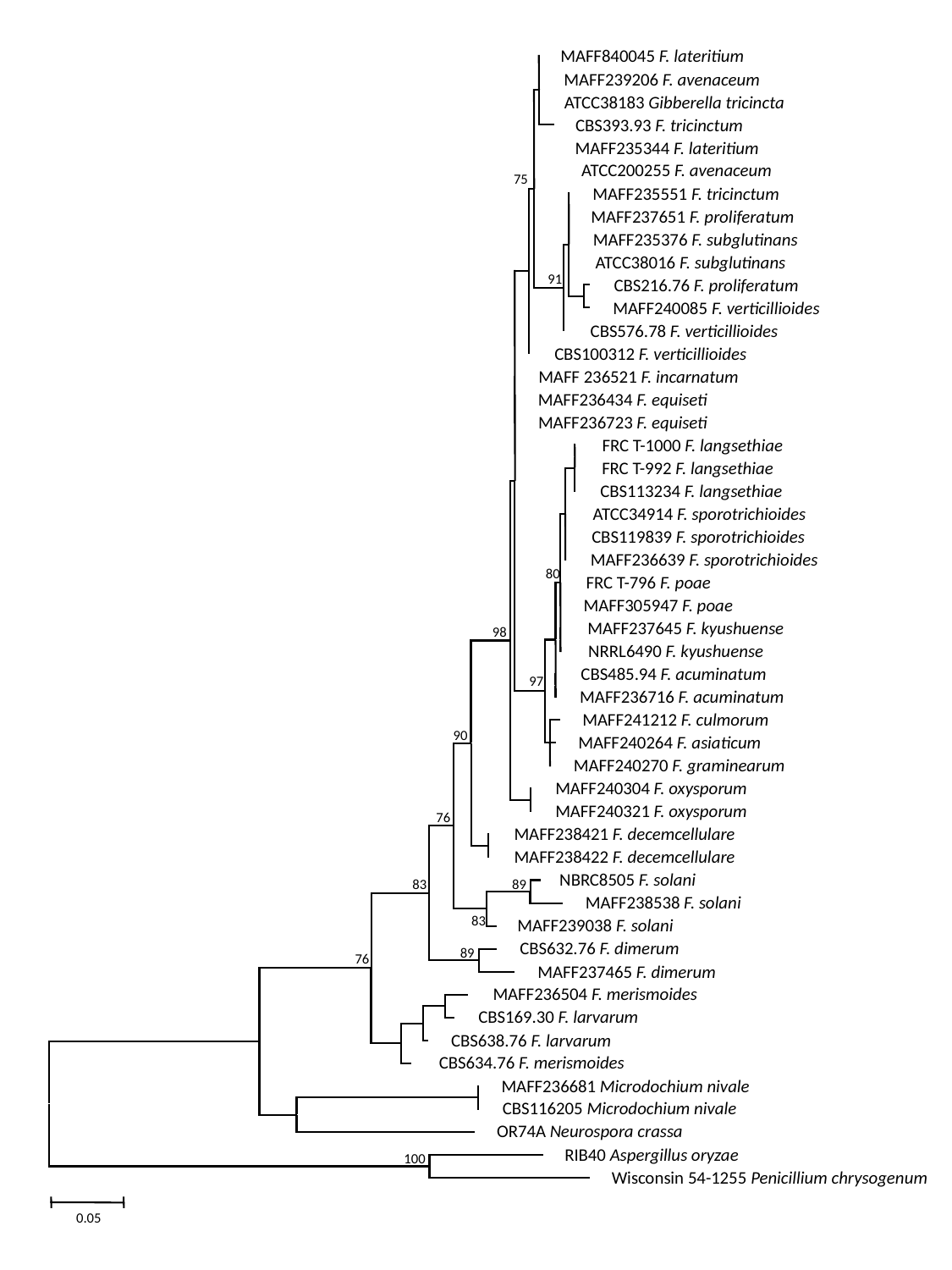

MAFF840045 F. lateritium
 MAFF239206 F. avenaceum
 ATCC38183 Gibberella tricincta
 CBS393.93 F. tricinctum
 MAFF235344 F. lateritium
 ATCC200255 F. avenaceum
 MAFF235551 F. tricinctum
 MAFF237651 F. proliferatum
 MAFF235376 F. subglutinans
 ATCC38016 F. subglutinans
 CBS216.76 F. proliferatum
 MAFF240085 F. verticillioides
 CBS576.78 F. verticillioides
 CBS100312 F. verticillioides
 MAFF 236521 F. incarnatum
 MAFF236434 F. equiseti
 MAFF236723 F. equiseti
 FRC T-1000 F. langsethiae
 FRC T-992 F. langsethiae
 CBS113234 F. langsethiae
 ATCC34914 F. sporotrichioides
 CBS119839 F. sporotrichioides
 MAFF236639 F. sporotrichioides
 FRC T-796 F. poae
 MAFF305947 F. poae
 MAFF237645 F. kyushuense
 NRRL6490 F. kyushuense
 CBS485.94 F. acuminatum
 MAFF236716 F. acuminatum
 MAFF241212 F. culmorum
 MAFF240264 F. asiaticum
 MAFF240270 F. graminearum
 MAFF240304 F. oxysporum
 MAFF240321 F. oxysporum
 MAFF238421 F. decemcellulare
 MAFF238422 F. decemcellulare
 NBRC8505 F. solani
 MAFF238538 F. solani
 MAFF239038 F. solani
 CBS632.76 F. dimerum
 MAFF237465 F. dimerum
 MAFF236504 F. merismoides
 CBS169.30 F. larvarum
 CBS638.76 F. larvarum
 CBS634.76 F. merismoides
 MAFF236681 Microdochium nivale
 CBS116205 Microdochium nivale
 OR74A Neurospora crassa
 RIB40 Aspergillus oryzae
Wisconsin 54-1255 Penicillium chrysogenum
75
91
80
98
97
90
76
89
83
83
89
76
100
0.05
