# Supplementary material for: Molecular phylogeny of the higher and lower taxonomy of the Fusarium genus and differences in the evolutionary histories of multiple genes
Source: BMC Evol Biol. 2011 Nov 3;11:322. doi: 10.1186/1471-2148-11-322 (PMC3270093; doi:10.1186/1471-2148-11-322)
Supplement: Additional file 6 — Supplementary method S2. [file 1471-2148-11-322-S6.DOC]

**Additional file 6 – Supplementary method S2.**

Because the non-synonymous substitution rate for *EF-1*αwas the slowest of all the sequences examined in this study (data not shown), it seems that *EF-1*αhas the minimal effect of multiple hits. For this reason, it was thought that we can assume that the distance of the non-synonymous substitutions of *EF-1*αis approximately proportional to the time. However, the most of the distances of intra-species or among closely related species became zero because of too slow substitution rate of this gene and too short length of the exon of this gene. Therefore, the non-synonymous substitution distance of *EF-1*αis not appropriate to use as the time axis. Therefore, we used the distances of the non-synonymous substitutions of β*-tub* as the x axis in Figure 2, which had the second slowest rate. To confirm the effect of multiple hits in nucleotide sequences of non-synonymous substitutions of β-tub, we used the distances of the non-synonymous substitutions of *EF-1*α as the x axis only in Figure 2A. The graph of the non-synonymous substitutions for β*-tub* is almost linear, indicating that these substitutions are not saturated.
