# Supplementary material for: Molecular phylogeny of the higher and lower taxonomy of the Fusarium genus and differences in the evolutionary histories of multiple genes
Source: BMC Evol Biol. 2011 Nov 3;11:322. doi: 10.1186/1471-2148-11-322 (PMC3270093; doi:10.1186/1471-2148-11-322)
Supplement: Additional file 7 — Supplementary method S3. [file 1471-2148-11-322-S7.DOC]

**Additional file 7 – Supplementary method S3.**

To evalate the corrective effect of the number of the multiple substitutions at the synonymous sites by the maximum likelihood method with the codon substitution model, the following procedures were applied. The numbers of the synonymous and non-synonymous substitutions per sites were estimated model by the CODEML program of PAML ver 4.4 [43]. The parameters were estimated from the each pairwise sequences. Nei and Gojobori's method [46] based on the p-distance was also applied to infer the unadjastive values using MEGA4. [47].
