# Supplementary figures and images for: Molecular phylogeny of the higher and lower taxonomy of the Fusarium genus and differences in the evolutionary histories of multiple genes
Source: BMC Evol Biol. 2011 Nov 3;11:322. doi: 10.1186/1471-2148-11-322 (PMC3270093; doi:10.1186/1471-2148-11-322)

## Slide 1
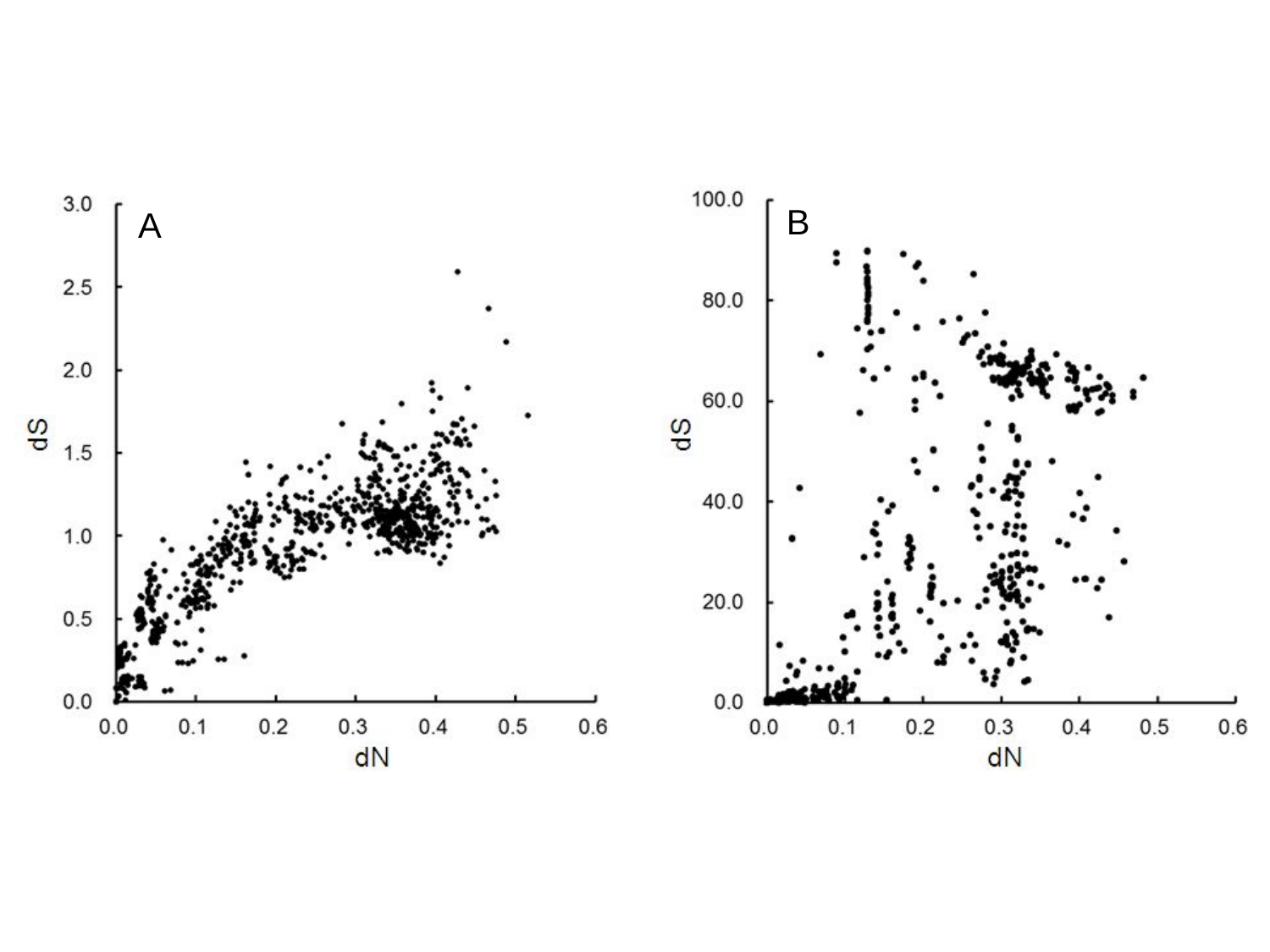

B
A

Supplement: Additional file 8 — Supplementary figure S5. Comparisons of the evolutionary distances by two methods. Number of non-synonymous substitutions per non-per synonymous site (dS). Panel A: Distance method modified Nei-Gojorbori(JC) k = 2.5; panel B: ML method the codon model (pairwise). The estimated synonymous substitution reached plateau from the analysis by Nei Gojobori method. However there was no such tendency from the analysis by the maximum likelihood method. It means that although there were many multiple substitutions at the synonymmous sites, the maximum likelihood effectively correct the numbers of the multiple substitutions. [file 1471-2148-11-322-S8.PPT]
